# Impact of Universal Nirsevimab Immunoprophylaxis on RSV-Related Hospitalizations in Infants: A Two-Season Multicenter Study in Northern Italy

**DOI:** 10.3390/pathogens15070698

**Published:** 2026-07-02

**Authors:** Nefer Roberta Gianotto, Neftj Ragusa, Virginia Deut, Chiara Mattivi, Marta Cherubini Scarafoni, Silvia Dominici, Giulia Mazzetti, Matteo Sandei, Chiara Lo Presti, Cenni Manuela, Mario Michele Calvo, Massimo Berger

**Affiliations:** 1 Department of Pediatrics and Neonatology, Ivrea Hospital, ASLTO4, 10015 Ivrea, Italy; 2 Department of Pediatrics and Neonatology, Ciriè Hospital, ASLTO4, 10073 Ciriè, Italy; 3 Department of Pediatrics and Neonatology, Chivasso Hospital, ASLTO4, 10034 Chivasso, Italy

**Keywords:** respiratory syncytial virus (RSV), Nirsevimab, infants

## Abstract

Respiratory syncytial virus (RSV) is the leading cause of bronchiolitis and hospitalization in infants worldwide. In 2024, the Piedmont region introduced universal immunoprophylaxis with Nirsevimab for all infants experiencing their first RSV season. We carried out a multicenter retrospective observational study across the three pediatric units of ASL TO4 (Ivrea, Ciriè, Chivasso), comparing bronchiolitis-related hospitalizations during the 2023–2024 season (pre-Nirsevimab) with those from the 2024–2025 season (post-Nirsevimab). The primary outcome was the proportion of RSV-positive hospitalizations. Secondary outcomes included age at admission, need for respiratory support, PICU/NICU transfer, and length of stay. Immunization coverage was assessed using the regional electronic registry. Immunization coverage exceeded 88% across all centers (overall 90.4%). A total of 179 bronchiolitis hospitalizations were recorded (134 pre- vs. 45 post-Nirsevimab). RSV-positive admissions showed a reduction from 70.9% to 55.6% after implementation (OR 0.52; 95% CI 0.24–1.09). Center-specific analyses suggested reductions in Ciriè (OR 2.48; 95% CI 1.41–4.39) and Chivasso (OR 2.28; 95% CI 1.09–4.77), with a similar trend observed in Ivrea. In a supplementary denominator-based analysis restricted to infants younger than 12 months, RSV-related hospitalization incidence decreased from 42.0 to 10.3 per 1000 infants between seasons (OR 4.23; 95% CI 2.64–6.78; *p* < 0.0001). Disease severity remained unchanged between seasons in terms of respiratory support, length of stay, and PICU/NICU transfers. Age at admission increased significantly during the post-intervention season (mean 118.3 vs. 160.9 days; Welch’s two-sample *t*-test, *p* = 0.026). Among 15 immunized infants hospitalized in 2024–2025, 6 were RSV-positive, none required intensive care, and only two needed high-flow nasal cannula (HFNC). Universal Nirsevimab prophylaxis was associated with a trend toward reduction in RSV-related hospitalizations at the aggregate level, although the overall comparison did not reach statistical significance. Center-specific analyses suggested reductions in RSV-positive admissions in some participating units. A supplementary denominator-based analysis among infants younger than 12 months showed a lower incidence of RSV-related hospitalizations during the post-implementation season. No evidence of increased severity among breakthrough cases was observed. High coverage demonstrated the feasibility of implementation and its potential public health value. Continued longitudinal surveillance over additional RSV seasons is essential to better define the durability of protection and long-term epidemiological impact.

## 1. Introduction

Bronchiolitis is a clinical condition marked by respiratory distress that predominantly affects infants within their first year of life. The illness typically starts with symptoms of the upper respiratory tract, such as nasal congestion, before progressing to involve the lower airways. In infants who are otherwise healthy, bronchiolitis is generally self-limited and resolved without major complications. Most mild presentations can be managed at home with supportive measures, including adequate hydration, nasal suctioning, and close observation for any signs of worsening [1]. Respiratory syncytial virus (RSV) represents the leading causative agent of bronchiolitis. Other pathogens that may contribute to the disease include rhinoviruses, influenza and parainfluenza viruses, as well as Mycoplasma pneumoniae [2]. Virologically, Human Respiratory Syncytial Virus (hRSV) is a single-stranded, negative-sense RNA virus belonging to the family Pneumoviridae and the genus Orthopneumovirus. Its linear, non-segmented genome is approximately 15.2 kb long and contains 10 genes encoding 11 proteins, including the fusion (F) and attachment (G) glycoproteins involved in viral entry and infectivity. RSV is classified into two major antigenic subtypes, A and B. Its circulation follows a seasonal pattern that varies according to the climate; in temperate regions of the Northern Hemisphere, transmission typically occurs from October to early May, with the highest incidence between December and February [3]. Globally, RSV represents the primary cause of acute lower respiratory tract infections (ALRTIs) in infants. Each year, it is responsible for an estimated 33.1 million ALRTI episodes in children under five, leading to roughly 3.2 million hospitalizations and more than 100,000 deaths worldwide [4,5]. The infection predominantly affects healthy newborns and young infants: 80–90% of hospitalized children are full-term infants without identifiable risk factors. Severe RSV bronchiolitis may require hospitalization and supportive management, including oxygen supplementation, hydration, and respiratory support in selected cases. Current guidelines do not recommend the routine use of corticosteroids, except in specific clinical circumstances or associated comorbidities [6]. Beyond the acute phase, RSV can also contribute to long-term respiratory issues, including recurrent wheezing and the development of asthma [7].

RSV infections are associated with significant healthcare costs. A recent national study of children aged 1 month to 1 year admitted to the IRCCS Bambino Gesù Children’s Hospital in Rome found that hospitalizations for RSV-related bronchiolitis were linked to higher average costs per patient (€5753.4 ± €2041.6) compared with bronchiolitis caused by non-RSV pathogens (€5395.2 ± €2040.9) (*p* = 0.04) [8].

Despite improvements in clinical care, RSV continues to cause substantial morbidity and mortality, with considerable economic and emotional repercussions for affected families. Currently, the main preventive strategy relies on the monoclonal antibody palivizumab. However, its use is limited to specific high-risk groups, such as infants born before 29 weeks of gestation, children with hemodynamically significant congenital heart disease, and those with pulmonary abnormalities, neuromuscular conditions that impair airway secretion clearance, or primary/secondary immunodeficiencies [9].

The use of palivizumab is restricted not only by its limited indications but also by its high cost, which hinders broad implementation. Furthermore, because of its short half-life, it must be administered monthly during peak RSV activity, reducing its practicality and accessibility [10,11].

This challenging scenario has been transformed by the introduction of Nirsevimab (Beyfortus, AstraZeneca and Sanofi), a long-acting monoclonal antibody against RSV that received approval from the European Medicines Agency (EMA) in October 2022 [12]. Nirsevimab is a recombinant human IgG1κ monoclonal antibody targeting the highly conserved antigenic site Ø of the prefusion RSV fusion (F) protein. By binding to this epitope, it inhibits viral fusion with host respiratory epithelial cells and prevents viral entry and cell-to-cell spread. Its prolonged half-life and strong neutralizing capacity against a wide array of RSV strains, including those resistant to other antibodies, offer meaningful advantages. A single dose can provide protection for at least five months throughout the RSV season, making it a promising option for universal passive immunoprophylaxis in newborns and infants [9].

The introduction of Nirsevimab has proven highly effective in reducing severe RSV bronchiolitis and related hospitalizations. In the Harmonie trial, only 0.3% of infants who received Nirsevimab were hospitalized within 180 days of life, compared with 1.7% in the standard care group, corresponding to an efficacy of 82.7% (*p* < 0.0001) [13].

Following EMA approval in October 2022, Nirsevimab was subsequently authorized in the United Kingdom (9 November 2022), Canada (19 April 2023), and the United States (17 July 2023) [6]. Several countries—such as France, Spain, the United States, and Luxembourg—have already adopted RSV prevention programs, reporting marked reductions in hospitalizations and Intensive Care Unit (ICU) admissions for RSV bronchiolitis [12,14,15,16]. In Italy, a passive immunoprophylaxis program began in the Valle d’Aosta region during the 2023–2024 season, resulting in zero hospitalizations for RSV-related lower respiratory tract infections among newborns who received Nirsevimab [5,9]. Based on these promising results, Nirsevimab has become part of routine clinical practice as of November 2024. Although several recent European and international real-world studies have demonstrated the effectiveness and safety of Nirsevimab in reducing RSV-related hospitalizations, important evidence gaps remain regarding its long-term epidemiological impact, regional implementation outcomes, breakthrough infections, and real-world effectiveness across different healthcare settings. In Italy, data are still limited, particularly at the regional level. In the Piedmont region of Northern Italy, the RSV prevention program was launched in November 2024. Nirsevimab was offered to all infants born during the RSV epidemic season (through March 2025), as well as to infants born between January and October 2024 who were expected to experience their first RSV season during autumn–winter 2024–2025.

The purpose of this study is to analyze initial real-world findings on the impact of Nirsevimab in reducing the number of bronchiolitis hospitalizations and the proportion of RSV-positive hospitalizations among infants in the Canavese region, a mountainous district located 50 km from the tertiary pediatric referral center, through a comparison of data from the 2023–2024 and 2024–2025 seasons.

## 2. Materials and Methods

### 2.1. Study Design and Setting

We conducted a multicenter retrospective observational study analyzing and comparing data from the three pediatric departments within ASLTO 4 (Ivrea, Ciriè, and Chivasso), serving the mountainous Canavese area with a population of over 500,000 inhabitants.

### 2.2. Study Population and Inclusion/Exclusion Criteria

The study population included all children under 24 months of age at the time of hospitalization with a diagnosis of bronchiolitis (caused by RSV or other agents) who were admitted for respiratory symptoms during the RSV epidemic season. Two epidemic seasons were compared: 2023–2024, prior to the introduction of Nirsevimab, and 2024–2025, after its implementation. For each season, the RSV epidemic period was defined as 1 November to 31 March of the following year, and hospitalizations occurring within this period were included. Although universal Nirsevimab prophylaxis targets infants during their first RSV season, the present study included all children under 24 months hospitalized for bronchiolitis during the study periods to assess the real-world burden of bronchiolitis hospitalizations before and after program implementation.

Respiratory symptoms used for screening purposes were defined as the presence of at least one respiratory sign or symptom (including cough, nasal congestion or rhinorrhea, sore throat, sneezing, shortness of breath or increased work of breathing, wheezing, tachypnea, or dyspnea), with or without fever. Bronchiolitis diagnosis was established independently by the attending pediatricians according to established pediatric guidelines and overall clinical assessment. None of the included patients had documented congenital or acquired immunodeficiency. Infants were excluded if they had no respiratory symptoms, suffered from sudden infant death syndrome, had a nosocomial infection (onset of respiratory symptoms more than two days after hospitalization), were hospitalized for less than 24 h before transfer to another facility (e.g., due to bed saturation for less severe or lower-risk cases), or if their parents had refused consent for the use of their personal health data.

### 2.3. Nirsevimab Administration and Coverage

Beginning 1 November 2024, Nirsevimab was made available in Piedmont to all infants born between 1 January 2024, and 31 March 2025. Infants born during the season were preferably administered passive immunoprophylaxis in hospital nurseries on the second day of life, whereas those born before the season received the prophylaxis from their family pediatricians in early November (catch-up administration), following standard procedures. Weekly immunization coverage data were available for the three maternity wards. Neonates transferred directly to the neonatal intensive care unit (NICU) after birth were excluded from the coverage calculation, as some were not discharged during the epidemic season. Coverage was calculated as the proportion of newborns receiving Nirsevimab in the maternity nurseries of the three participating ASL TO4 centers during the 2024–2025 RSV season. The denominator was defined as the total number of live births occurring in the same maternity wards during the study period, excluding neonates transferred directly to tertiary-level NICUs. This estimate represents a nursery-based coverage measure. Catch-up immunization administered in the outpatient setting was recorded in absolute numbers; however, the number of eligible infants in this cohort could not be reliably reconstructed across centers and was therefore not included in the coverage calculation. Reasons for non-administration in the maternity wards were not systematically recorded in a structured manner and could not be disaggregated (e.g., parental refusal versus early NICU transfer).

### 2.4. Outcomes

The primary outcome was the proportion of RSV-positive bronchiolitis hospitalizations during the two study seasons. Secondary outcomes reflecting disease severity included the need for respiratory support, classified according to the highest level required during hospitalization: low-flow oxygen, high-flow nasal cannula (HFNC), continuous positive airway pressure (CPAP) or helmet ventilation, and endotracheal intubation. Admissions to the neonatal/pediatric intensive care unit (NICU/PICU) and length of hospital stay were also evaluated.

### 2.5. Data Collection

All electronic medical records of bronchiolitis-associated hospitalizations were manually reviewed to extract demographic, clinical, and treatment data. Collected information included vital signs, clinical observations, respiratory support (non-invasive or invasive), feeding support (enteral or parenteral), NICU/PICU admissions, age, gender, birth weight, gestational age (preterm defined as <37 weeks), comorbidities, previous respiratory infections, and the presence of siblings in the household.

All patients underwent RSV testing using the BinaxNOW RSV Card rapid antigen assay (Abbott), which represented the standard diagnostic method across the three participating centers. In selected cases, including suspected coinfections or atypical clinical evolution, molecular confirmation was requested and respiratory specimens were referred to the regional reference laboratory in Turin for polymerase chain reaction (PCR) testing. Overall, 24 of 179 hospitalized patients (13.4%) underwent PCR confirmation. For infants hospitalized during the 2024–2025 season, Nirsevimab administration was determined by (i) inspecting electronic birth records (where immunization status was recorded if administered prior to discharge) and (ii) reviewing the electronic medical records of RSV-associated hospitalizations, with parental confirmation when needed.

Infants hospitalized with an RSV-associated lower respiratory tract infection (LRTI) during the 2024–2025 season were stratified into immunized (Nirsevimab administered) and non-immunized (no Nirsevimab) groups. Disease characteristics, including age at admission, sex, preterm birth status, need for supplemental oxygen or feeding support, and PICU admission, were compared between the two groups.

### 2.6. Ethics

The study was conducted in accordance with the Declaration of Helsinki and was approved by the Competent Territorial Ethics Committee (code: DGR 7-143/2024/XII; approval date: 16 September 2024). Data were retrospectively collected from routinely recorded clinical information and analyzed in anonymized form. Authorization for the use of health data was covered through the regional Health File consent procedure, according to local regulatory requirements. No additional study-specific written informed consent was required.

### 2.7. Data Management

Data was obtained from six independent datasets, each corresponding to a specific hospital center and admission season. These datasets were merged into a single database to increase sample size, and two additional variables were created to indicate the admitting center and the season of hospitalization.

### 2.8. Statistical Analysis

The statistical analysis was primarily exploratory and descriptive in nature, aimed at comparing demographic and clinical characteristics between epidemic seasons. No multivariable adjustment was performed; therefore, observed associations should be interpreted cautiously, as they may have been influenced by unmeasured or residual confounding factors. Center- and time-stratified analyses were also considered exploratory due to the limited number of events within subgroups and the reduced statistical power for these comparisons.

Descriptive statistics were generated for demographic and clinical variables. Differences between the pre- and post- seasons were assessed using appropriate statistical tests based on variable type and distribution. Differences in proportions (e.g., RSV positivity, use of respiratory support, PICU admissions) were evaluated using Pearson’s chi-square test with Yates’ continuity correction or Fisher’s exact test when expected frequencies were low. Differences in continuous variables were assessed according to their distribution. Comparisons of age at hospitalization and mean length of hospital stay between seasons were performed using Welch’s two-sample *t*-test. For continuous variables not normally distributed, including duration of oxygen or ventilatory support, comparisons between seasons were performed using the Wilcoxon rank-sum test with continuity correction. Statistical significance was set at *p* < 0.05. Additionally, for each hospital center, odds ratios (ORs) were calculated to quantify differences in the proportion of RSV-positive hospitalizations between the 2023–2024 and 2024–2025 seasons. As a supplementary denominator-based analysis, RSV-related hospitalization incidence was estimated computing infants younger than 12 months of age during each RSV season as the denominator. The denominator was reconstructed from the number of live births recorded in the three participating centers during the corresponding birth periods and included all infants who were younger than 12 months during the corresponding RSV season and therefore potentially at risk of RSV hospitalization. Specifically, for the 2023–2024 season, the denominator comprised infants born between 1 November 2022, and 31 October 2023, whereas for the 2024–2025 season it comprised infants born between 1 January 2024, and 31 March 2025, corresponding to infants eligible to experience their first RSV season during the implementation of the universal Nirsevimab program. The numerator consisted of RSV-positive bronchiolitis hospitalizations among infants younger than 12 months during each season. Incidence rates were expressed per 1000 infants and compared between seasons by calculating odds ratios with 95% confidence intervals. The raw denominators, numerators, and incidence estimates used for this supplementary analysis are reported in Supplementary Table S1. The denominator was reconstructed using the number of live births recorded in the maternity units of the three participating hospitals rather than population-based birth registries. The same denominator construction, inclusion criteria, and analytical approach were applied consistently across both RSV seasons and all participating centers, ensuring internal consistency of the comparison. Therefore, this supplementary analysis should be interpreted as a hospital network-based estimate of RSV hospitalization burden rather than a population-based incidence estimate.

## 3. Results

High levels of Nirsevimab uptake were achieved across the ASLTO4 district during the 2024–2025 season, indicating successful implementation of the prophylaxis program. Coverage exceeded 88% in all three participating centers, reaching 91.7% in Chivasso, 90.7% in Ivrea, and 88.7% in Ciriè, with an overall Nirsevimab administration rate of 90.4% among eligible newborns. This estimate refers to nursery-based administration only and excludes NICU transfers and outpatient catch-up immunization, which were not included in the formal denominator due to lack of consistent eligibility data.

**Figure 1:**
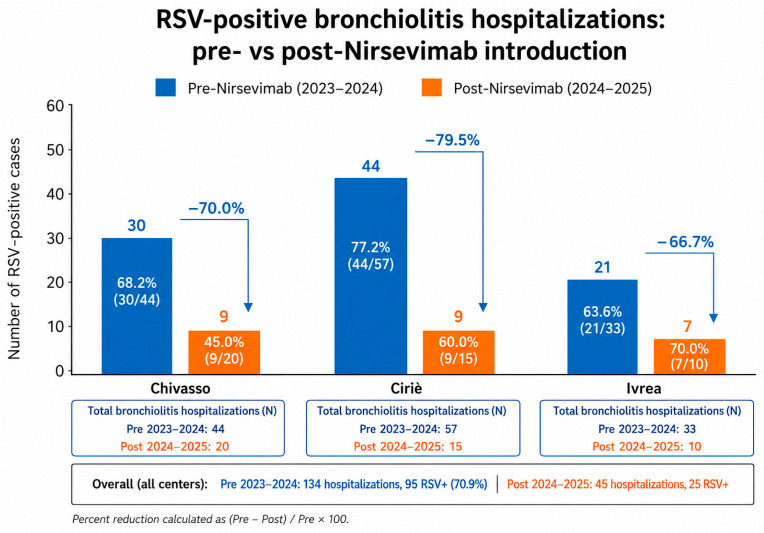
RSV-positive bronchiolitis hospitalizations in the pre-Nirsevimab (2023–2024) and post-Nirsevimab (2024–2025) seasons across the three ASL TO4 pediatric centers.

**Figure 2:**
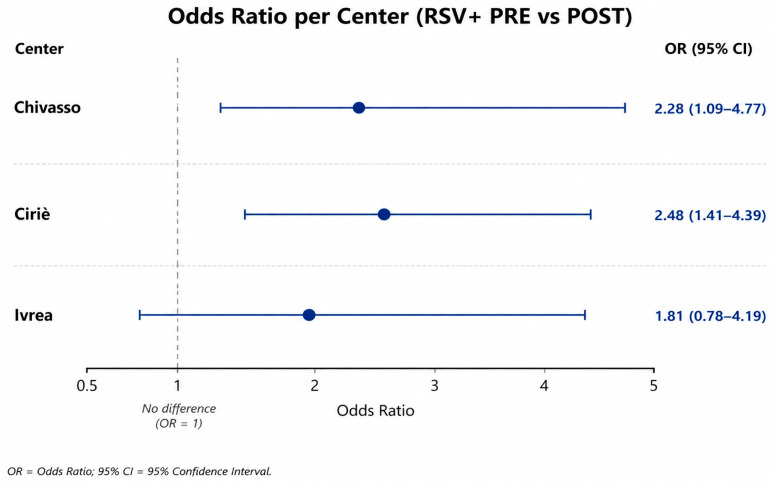
Odds ratios and 95% confidence intervals for RSV-positive bronchiolitis hospitalizations in the pre-Nirsevimab versus post-Nirsevimab seasons across the three ASL TO4 pediatric centers.

A total of 179 hospitalizations for bronchiolitis were recorded across the three pediatric units of ASL TO4 over the two study seasons: 134 in 2023–2024 (pre-Nirsevimab) and 45 in 2024–2025 (post-Nirsevimab). Their main features, stratified by period, are summarized in Table 1. Since the study included all bronchiolitis hospitalizations under 24 months of age, the observed population partially overlaps with the regional target cohort for Nirsevimab immunoprophylaxis, which mainly comprised infants experiencing their first RSV season. The proportion of RSV-positive cases decreased from 70.9% in the pre-season to 55.6% in the post-intervention season, with the most substantial decrease observed in Ciriè, followed by Chivasso and Ivrea (Figure 1). Although this reduction did not reach conventional statistical significance (χ^2^ = 2.93, *p* = 0.087; Fisher’s exact test *p* = 0.068), the estimated odds ratio suggested a possible decrease in RSV positivity after the introduction of Nirsevimab (OR = 0.52; 95% CI 0.24–1.09). Stratified analysis by center showed heterogeneous patterns in RSV positivity between the two seasons. Odds ratios were calculated using the 2024–2025 season (post-intervention period) as the reference category; therefore, OR values > 1 indicate a higher likelihood of RSV hospitalization during the 2023–2024 season (pre-intervention period). In Chivasso, the odds ratio was 2.28 (95% CI 1.09–4.77), corresponding to a lower proportion of RSV-positive bronchiolitis hospitalizations in the 2024–2025 season compared with 2023–2024. In Ciriè, a similar pattern was observed (OR 2.48; 95% CI 1.41–4.39), while in Ivrea the difference between seasons was less pronounced and did not reach statistical significance (OR 1.81; 95% CI 0.78–4.19). A graphical representation of the center-level odds ratios and confidence intervals is provided in Figure 2.

Overall, these center-specific estimates should be interpreted cautiously given the limited number of events within each subgroup and the exploratory nature of these analyses. The observed heterogeneity across centers may reflect differences in sample size, local epidemiology, and healthcare utilization patterns rather than true differences in the effect of Nirsevimab implementation. Absolute numbers of RSV-positive hospitalizations followed a similar pattern across centers, with a reduction observed in the post-implementation season, although statistical inference at the center level is limited by small sample sizes.

To better evaluate the impact of Nirsevimab within the population most relevant to the immunoprophylaxis program, an additional supplementary denominator-based analysis was performed using infants younger than 12 months of age during each RSV season as the denominator. The incidence of RSV-related hospitalization decreased from 42.0 per 1000 infants in the 2023–2024 season to 10.3 per 1000 infants in the 2024–2025 season (Supplementary Table S1). The corresponding odds ratio was 4.23 (95% CI 2.64–6.78; *p* < 0.0001), indicating a substantially lower hospitalization burden during the post-implementation season.

Age-stratified analyses showed that RSV-positive bronchiolitis hospitalizations were concentrated in infants younger than 6 months across both seasons. A reduction in RSV positivity was observed in most age groups in the post-implementation season, although estimates in older age categories were limited by small sample size. These findings suggest that the observed reduction in RSV-positive hospitalizations was consistent across key demographic subgroups but should be interpreted cautiously given the descriptive nature of the analyses and the limited number of events in several strata. Age-stratified results are reported in Table 2.

No statistically significant differences were observed between the two seasons for the secondary outcomes analyzed, except for a trend in RSV positivity.

**Figure 3:**
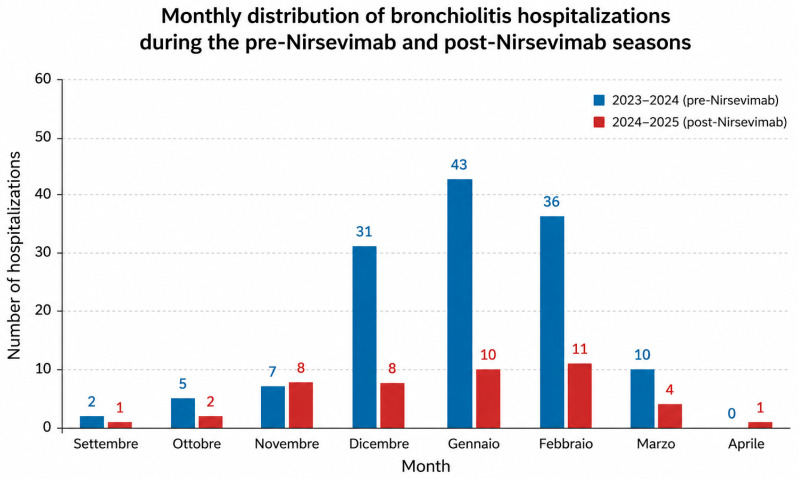
Monthly distribution of bronchiolitis hospitalizations during the pre-Nirsevimab season (2023–2024) and post-Nirsevimab season (2024–2025). Exact monthly hospitalization counts are displayed within the graph.

The length of hospitalization was comparable across periods (median 3.5 days in 2023–2024 vs. 3 days in 2024–2025; Wilcoxon *p* = 0.446), indicating that the introduction of Nirsevimab did not modify clinical severity in terms of length of stay. Similarly, admission to NICU/PICU remained rare and stable over time (5.3% vs. 4.4%; OR = 0.84; 95% CI 0.08–4.63; Fisher *p* = 1), with no evidence of differences between seasons. The proportion of patients requiring respiratory support was also unchanged (72.4% vs. 71.1%; Fisher *p* = 0.85), as was the duration of oxygen therapy (mean 4.1 vs. 3.9 days; Wilcoxon *p* = 0.528), suggesting similar clinical severity across both periods. A significant increase in age at hospitalization was observed in 2024–2025 compared with the previous season (mean 118.3 ± 104.3 vs. 160.9 ± 126.3 days; Welch’s two-sample *t*-test, *p* = 0.026). This shift suggests a delayed onset of clinically relevant diseases among hospitalized infants following the introduction of prophylaxis. However, stratified analysis showed that this difference was limited to non-immunized infants, while age at hospitalization among those who received Nirsevimab remained comparable to the pre- period. Finally, the temporal distribution of admissions also differed between seasons (Figure 3). In 2023–2024, hospitalizations peaked sharply between December and January, whereas the 2024–2025 season showed a flatter pattern with substantially lower monthly counts across all corresponding months. This reduction aligned with the overall decline in the observed number of RSV-positive hospitalizations after the implementation of Nirsevimab. Overall, secondary outcomes indicate stable clinical severity across seasons, with a notable change only in age at hospitalization and no significant variations in supportive care parameters.

During the 2024–2025 season, 15 infants who had received Nirsevimab passive immunoprophylaxis were subsequently hospitalized for bronchiolitis. Among these cases, 6 (40%) tested positive for RSV, while the remaining 9 (60%) were RSV-negative. Notably, none of the RSV-positive infants required transfer to the neonatal or pediatric intensive care unit, and only 2 of them required respiratory support with High-Flow Nasal Cannula (HFNC), indicating a generally mild clinical course despite breakthrough infection.

## 4. Discussion

In this multicenter observational study conducted across three pediatric units of ASL TO4, we observed a reduction in the proportion of RSV-positive bronchiolitis hospitalizations and in the absolute number of RSV-positive admissions during the 2024–2025 season following the introduction of universal Nirsevimab immunoprophylaxis. However, the overall reduction in RSV-positive hospitalizations did not reach conventional statistical significance in the pooled analysis (OR 0.52; 95% CI 0.24–1.09). Nevertheless, the direction and magnitude of the observed effect were consistent with the efficacy demonstrated in randomized trials—particularly the HARMONIE study—and with emerging real-world evidence from early implementation programs in Europe and the United States [12,13,16,17]. Therefore, our findings should be interpreted as suggesting a possible reduction in RSV-related hospitalization burden following Nirsevimab implementation rather than demonstrating a statistically significant effect in this study population.

The decline in RSV-positive admissions was observed across all centers, although the magnitude varied. Chivasso and Ciriè showed a lower proportion of RSV-positive bronchiolitis hospitalizations in the post-intervention season compared with the previous season (OR 2.28, 95% CI 1.09–4.77; OR 2.48, 95% CI 1.41–4.39, respectively), while Ivrea showed a similar direction of effect without a clear difference between seasons (OR 1.81, 95% CI 0.78–4.19), likely attributable to smaller sample size. In a supplementary denominator-based analysis restricted to infants younger than 12 months during each RSV season, the incidence of RSV-related hospitalization decreased from 42.0 to 10.3 per 1000 infants between seasons (OR 4.23; 95% CI 2.64–6.78; *p* < 0.0001). Although this analysis should be interpreted in the context of the study’s observational design, the use of identical denominator construction and inclusion criteria across both study seasons supports the internal consistency of the comparison. Nevertheless, because the denominator was reconstructed from births occurring in the participating maternity units rather than the entire regional infant population, these estimates should be interpreted as reflecting hospitalization burden within the study network rather than true population-based incidence. Importantly, despite the overall decrease in case numbers, the severity of illness among hospitalized infants remained comparable between seasons, with no differences in the need for respiratory support, length of stay, or intensive care transfers. These findings indicate that Nirsevimab predominantly reduced the observed burden of RSV-positive hospitalizations rather than modifying the clinical trajectory once hospitalization occurs, aligning with previous evidence that passive immunization prevents infection rather than attenuating established disease. While severity indicators remained stable across seasons, the low frequency of severe outcomes may have reduced the ability of the study to detect moderate but clinically relevant differences. Larger multicenter studies will be needed to better define the impact of Nirsevimab on RSV disease severity among hospitalized infants.

Breakthrough cases were uncommon, representing a small proportion of all hospitalizations, and their clinical course was generally mild. Among the six RSV-positive infants who had received Nirsevimab, none required PICU transfer and only two required HFNC support. Although these observations suggest a generally mild clinical course among breakthrough RSV infections, the very limited sample size and the descriptive nature of this subgroup analysis preclude any definitive conclusions regarding disease severity in immunized infants. The high immunization coverage achieved across centers in our study, together with the similarly high levels reported in the post-Nirsevimab administration studies from other settings, underscores the broad acceptability of Nirsevimab, the feasibility of its large-scale implementation, and its likely contribution to reducing RSV transmission and the associated hospitalization burden [12,17].

Although real-world data on Nirsevimab are increasing, comparative analyses across consecutive pre- and post- seasons remain scarce in Europe and particularly in Italy. A notable exception is the nationwide study from Luxembourg, which reported a 69% reduction in RSV-associated hospitalizations among infants < 6 months of age when comparing the 2022–2023 and 2023–2024 seasons following the introduction of Nirsevimab [17]. Unlike those population-based studies, our analysis was based on hospitalized bronchiolitis cases and was not designed to estimate population-level RSV hospitalization incidence, as denominators for the eligible infant population were not consistently available across centers. In Italy, evidence is still emerging: a regional analysis from Tuscany compared the 2024–2025 season with the three preceding seasons, showing a substantial decline in RSV-related outcomes, while another multicenter study from Central Italy directly contrasted the 2023–2024 and 2024–2025 seasons, further supporting a possible beneficial association between universal prophylaxis and reduced RSV hospitalization burden [1,18]. Studies adopting this design are essential to quantify the true effect of the intervention while accounting for seasonal variability and differences in timing of implementation. Our findings therefore contribute to the still limited national evidence base and highlight the need for continued longitudinal monitoring. Unlike population-based surveillance studies, our analysis was hospital-based and did not include complete denominators or person-time estimates for the underlying infant population. Therefore, the findings should be interpreted as changes in observed hospitalization burden and RSV positivity among hospitalized bronchiolitis cases rather than direct estimates of RSV hospitalization incidence or population-level risk reduction. To partially address this limitation, we performed a supplementary denominator-based analysis restricted to infants younger than 12 months, representing the age group most closely aligned with the target population for universal Nirsevimab prophylaxis.

In addition to the clinical implications, recent modeling and real-world studies have suggested that reductions in RSV-related hospitalizations following Nirsevimab implementation may translate into meaningful decreases in healthcare costs, particularly by reducing bed occupancy and emergency admissions [19,20].

Although a formal cost-effectiveness analysis was beyond the scope of this study, these findings support the broader public health value of universal RSV prophylaxis and highlight the importance of maintaining high coverage over subsequent seasons.

An increase in the median age at hospitalization during the post-season was also observed. This finding may reflect several non-mutually exclusive mechanisms. First, the selective protection conferred by Nirsevimab in younger infants may have reduced hospitalizations in the most vulnerable age group, thereby shifting the age distribution toward older infants who were not eligible for or did not receive prophylaxis. This shift has been documented in other cohorts and may reflect delayed susceptibility in immunized infants, changes in viral circulation dynamics, or reduced early-season exposure. Second, reduced RSV circulation following the introduction of immunoprophylaxis may have contributed to delayed viral exposure, resulting in infections occurring later in infancy. Finally, changes in community transmission dynamics and potential indirect (herd) effects may have influenced the timing and distribution of RSV infections. These hypotheses remain speculative, as viral sequencing and detailed transmission data were not available in this study. Further multicenter studies are needed to clarify the underlying mechanisms [16,18]. While the clinical implications of this trend remain uncertain, it may influence the timing of future immunization strategies and warrants continued surveillance across subsequent seasons.

This study has several limitations. Its observational design precludes causal inference, and residual confounding cannot be excluded. We compared only two consecutive seasons, which differed in circulating viral patterns, and RSV surveillance relied on routine clinical testing rather than systematic screening. The sample size, particularly for center-specific and subgroup analyses, limits statistical power. In addition, the comparison was based on only two consecutive RSV seasons, which may have reduced the ability to detect differences in some outcomes and limited the assessment of seasonal variability. Longer-term surveillance over additional RSV seasons will be needed to better evaluate the durability of protection and the sustained epidemiological impact of universal Nirsevimab immunoprophylaxis on RSV-related hospitalization burden and disease severity. Furthermore, complete population-based denominators and person-time estimates were not available for all participating centers. Consequently, the study was not designed to estimate RSV hospitalization incidence rates or population-level risk reduction. Because the study population was broader than the regional immunoprophylaxis target population, the findings should be interpreted as changes in observed hospitalization burden rather than direct estimates of vaccine effectiveness in the eligible cohort.

Additionally, our data reflects a single geographic area, which may reduce generalizability. RSV diagnosis was primarily based on the BinaxNOW RSV Card rapid antigen assay (Abbott), which was routinely used across all three participating centers. PCR confirmation was performed in a subset of patients (24/179, 13.4%), mainly in cases of suspected coinfection or atypical clinical evolution, with samples referred to the regional reference laboratory in Turin. Because rapid antigen tests and PCR assays differ in diagnostic sensitivity, some degree of misclassification of RSV status cannot be excluded, particularly in patients with lower viral loads. However, the use of a common diagnostic approach across centers likely limited differential ascertainment between study sites and seasons. Finally, we were unable to determine the prophylactic coverage of infants who received Nirsevimab in outpatient settings (the catch-up group).

Nonetheless, the strengths of the study include complete population capture across three hospitals, high immunization coverage, and the ability to compare consecutive pre- and post-implementation seasons within the same healthcare network.

## 5. Conclusions

In conclusion, this study observed a trend toward a lower burden of RSV-positive hospitalizations and bronchiolitis admissions following the implementation of universal Nirsevimab passive immunoprophylaxis in the ASL TO4 region. However, the overall reduction in RSV-positive hospitalizations did not reach conventional statistical significance in the primary pooled analysis and should therefore be interpreted with caution. No evidence of increased disease severity among breakthrough cases was observed. These findings suggest a possible beneficial impact of Nirsevimab implementation in routine clinical practice, while continued monitoring across future RSV seasons will be essential to better define the durability of protection, potential shifts in age distribution, and the long-term epidemiological impact of the intervention.

## Figures and Tables

**Table 1:** Baseline and clinical characteristics of hospitalized infants during the pre- and post-Nirsevimab seasons.

Variable	Level	PRE (n = 134)	POST (n = 45)	*p*-Value
**Study center, n (%)**	Ivrea	33 (24.6)	11 (24.4)	0.934
	Chivasso	38 (28.4)	14 (31.1)	
	Ciriè	63 (47.0)	20 (44.4)	
**Age at hospitalization, mean (SD), days**	—	118.34 (104.28)	160.93 (126.28)	**0.026**
**Sex, n (%)**	Male	63 (47.0)	18 (40.0)	0.413
	Female	71 (53.0)	27 (60.0)	
**Ethnicity, n (%)**	Caucasian	119 (88.8)	35 (77.8)	0.065
	Other	15 (11.2)	10 (22.2)	
**Siblings, n (%)**	No	22 (17.1)	7 (15.9)	0.861
	Yes	107 (82.9)	37 (84.1)	
**Body weight, mean (SD), g**	—	5781.11 (1900.95)	6650.47 (2077.39)	**0.013**
**Mechanical ventilation, n (%)**	No	128 (96.2)	45 (100.0)	0.187
	Yes	5 (3.8)	0 (0.0)	
**Length of hospital stay, mean (SD), days**	—	5.63 (2.48)	5.42 (2.58)	0.636
**RSV-positive bronchiolitis, n (%)**	No	39 (29.1)	20 (44.4)	0.058
	Yes	95 (70.9)	25 (55.6)	
**Duration of oxygen therapy, mean (SD), days**	—	4.11 (2.43)	3.94 (2.77)	0.743
**Highest level of respiratory support, n (%)**	Low-flow oxygen	68 (68.7)	22 (68.8)	0.997
	HFNC	19 (19.2)	6 (18.8)	
	Low-flow oxygen + HFNC	12 (12.1)	4 (12.5)	
**PICU/NICU admission, n (%)**	No	126 (94.7)	43 (95.6)	0.828
	Yes	7 (5.3)	2 (4.4)	

Abbreviations: SD, standard deviation; HFNC, high-flow nasal cannula; PICU, Pediatric Intensive Care Unit; NICU, Neonatal Intensive Care Unit; RSV, respiratory syncytial virus.

**Table 2:** Distribution of bronchiolitis according to age.

Age Group	Bronchiolitis 2023–2024, n	RSV-Positive 2023–2024, (%)	Bronchiolitis 2024–2025, n	RSV-Positive 2024–2025, (%)	OR (95% CI)	*p*-Value
<3 months	65	41 (63.1)	16	8 (50.0)	1.70 (0.56–5.10)	0.330
3–6 months	47	36 (76.6)	15	7 (46.7)	3.74 (1.10–12.60)	**0.050**
6–12 months	16	12 (75.0)	11	7 (63.6)	1.71 (0.32–9.10)	0.670
>12 months	6	6 (100.0)	3	3 (100.0)	NE †	1.000
**Total**	**134**	**95 (70.9)**	**45**	**25 (55.6)**	**1.94 (0.97–3.90)**	**0.058**

Abbreviations: RSV, respiratory syncytial virus; OR odds ratio; CI, confidence interval; NE, not estimable. † Odds ratio could not be estimated because all hospitalized infants in both seasons were RSV-positive within this age group. † NE (Not Estimable): the odds ratio could not be reliably estimated because all bronchiolitis cases in this age group were RSV-positive in both seasons (100% event rate), resulting in zero cells in the contingency table.

## Data Availability

The original contributions presented in this study are included in the article and Supplementary Material. Further inquiries can be directed to the corresponding author.

## Supplementary Materials

The following supporting information can be downloaded at: https://www.mdpi.com/article/10.3390/pathogens15070698/s1, Table S1: Population-based supplementary analysis of RSV-positive hospitalizations among infants younger than 12 months during the two RSV seasons.
