# Supplementary material for: Impact of Universal Nirsevimab Immunoprophylaxis on RSV-Related Hospitalizations in Infants: A Two-Season Multicenter Study in Northern Italy
Source: Pathogens. 2026 Jul 2;15(7):698. doi: 10.3390/pathogens15070698 (PMC13415040; doi:10.3390/pathogens15070698)
Supplement: Supplementary file 1 [file pathogens-15-00698-s001.zip › pathogens-4322671-supplementary.pdf]

Supplementary Table S1. Population-based supplementary analysis of RSV-positive hospitalizations among infants younger than 12 months during the two RSV seasons.

RSV season	Birth period included in denominator	Live births (n)	RSV-positive hospitalizations <12 months (n)	Incidence (per 1,000 infants)
2023–2024	1 November 2022 – 31 October 2023	2,117	89	42.0
2024–2025	1 January 2024 – 31 March 2025	2,141	22	10.3
